# Evaluation of a Monte Carlo based EPID system for patient‐specific IMRT and VMAT quality assurance

**DOI:** 10.1002/acm2.70178

**Published:** 2025-07-15

**Authors:** Wuji Sun, Chao Ge, Yinghua Shi, Xiaohe He, Li Xiao, Yanfang Liu, Zhi Wang, Tianlong Ji, Huidong Wang

**Affiliations:** ^1^ Department of Radiation Oncology & Therapy The First Hospital of Jilin University Changchun China; ^2^ United Imaging Healthcare Co., Ltd. Shanghai China; ^3^ Department of Radiation Oncology First Affiliated Hospital of Anhui Medical University Hefei China; ^4^ Department of Radiation Oncology The First Hospital of China Medical University Shenyang China; ^5^ Jilin Provincial Key Laboratory of Radiation Oncology & Therapy Department of Radiation Oncology & Therapy The First Hospital of Jilin University Changchun China; ^6^ NHC Key Laboratory of Radiobiology School of Public Health Jilin University Changchun China

**Keywords:** dosimetry, EPID, Monte Carlo, patient specific quality assurance, radiotherapy

## Abstract

**Purpose:**

This study aims to evaluate the performance of a new electronic portal imaging device (EPID) system with a Monte Carlo (MC) based algorithm for patient‐specific quality assurance (PSQA), ensuring its reliability and effectiveness in treatment verification efficiency and precision.

**Methods:**

Sixteen patients with various tumor sites were divided evenly into two groups for dynamic intensity‐modulated radiation therapy and volumetric modulated arc therapy verification. Measurements were performed on a UIH uRT‐linac 506c linear accelerator with a Varex Imaging XRD 1642 EPID. The performance of the EPID was assessed for sensitivity to errors in monitor units, collimator angle, field offset, field size, and multi‐leaf collimator functionality. Treatment plans were modified for each error and verified to determine the capability of the system to detect perturbations from the planned dose distribution, which was quantitatively analyzed using γ index analysis (2%/2 mm, 10% low‐dose threshold). Additionally, the ArcCHECK phantom was employed to validate the EPID system for PSQA and induced error detection. Pearson's correlation coefficient was employed to assess correlations between γ passing rates and induced errors.

**Results:**

The EPID system showed high accuracy in dose linearity and error detection, with γ passing rates consistently above 95% for original plans. Sensitivity tests indicated strong correlations between induced errors and γ passing rates, confirming the capability of this system to detect subtle dosimetric discrepancies and supporting its application for PSQA. Both EPID and ArcCHECK demonstrated comparable γ passing rates in PSQA of standardized plans and treatment plans, with EPID showing higher or comparable sensitivity to tested errors.

**Conclusion:**

The EPID Plan QA system showed promising accuracy and sensitivity in detecting errors, proving to be a reliable tool for PSQA. Its seamless integration into clinical workflows is expected to enhance treatment verification efficiency and ensure precise radiotherapy delivery.

## 1 INTRODUCTION

In recent years, many advanced treatment delivery techniques have been introduced such as intensity‐modulated radiation therapy (IMRT) and volumetric arc therapy (VMAT). The advent of stereotactic body radiation therapy, which delivers higher doses per fraction, has necessitated higher standard in dosimetric precision. This need has spurred the development and widespread adoption of reliable patient‐specific quality assurance (PSQA) tools.^1^, ^2^, ^3^, ^4^

Electronic portal imaging devices (EPIDs) have been used in clinical settings for decades and have become an integral part of medical linear accelerators.^5^, ^6^, ^7^ EPID serves two primary purposes in clinical treatment, verifying patient setup through in vivo imaging before or during radiation therapy, and validating treatment plans through dose verification.^8^, ^9^ Besides, EPID can also be used for quality assurance and quality control tasks, such as beam check, isocenter verification, treatment couch verification, and multi‐leaf collimator (MLC) tests.^8^ Compared to other QA devices, such as films, 2D diode and ion chamber (IC) arrays, EPID offers several advantages, including a denser array of measurement points, improved operational efficiency, and ease of use.^10^, ^11^ The advent of amorphous silicon (a‐Si) flat panel technology has led to improved image quality and dosimetric utility, making EPIDs a standard tool for pre‐treatment quality assurance by facilitating accurate dose verification.^12^, ^13^, ^14^

The American Association of Physicists in Medicine (AAPM) has provided guidance on the use of EPIDs on PSQA through Task Group Report 58 (TG‐58) and Task Group Report 307 (TG‐307).^9^, ^15^ EPID‐based PSQA can be performed either directly at the EPID level using a virtual slab phantom or within the patient/phantom using forward or backward projection methods.^15^ Forward projection methods compare the measured images or dose distributions with predicted ones at the EPID level, while back projection methods compare them in a phantom or patient. Such comparisons are generally made using γ index analysis resulting γ passing rates.^16^ γ index analysis can be provided by various software products. Therefore, an EPID system typically represents the combination of EPID imaging system and compatible software, used for dose measurements and dosimetric analyses, respectively.

**FIGURE 1:**
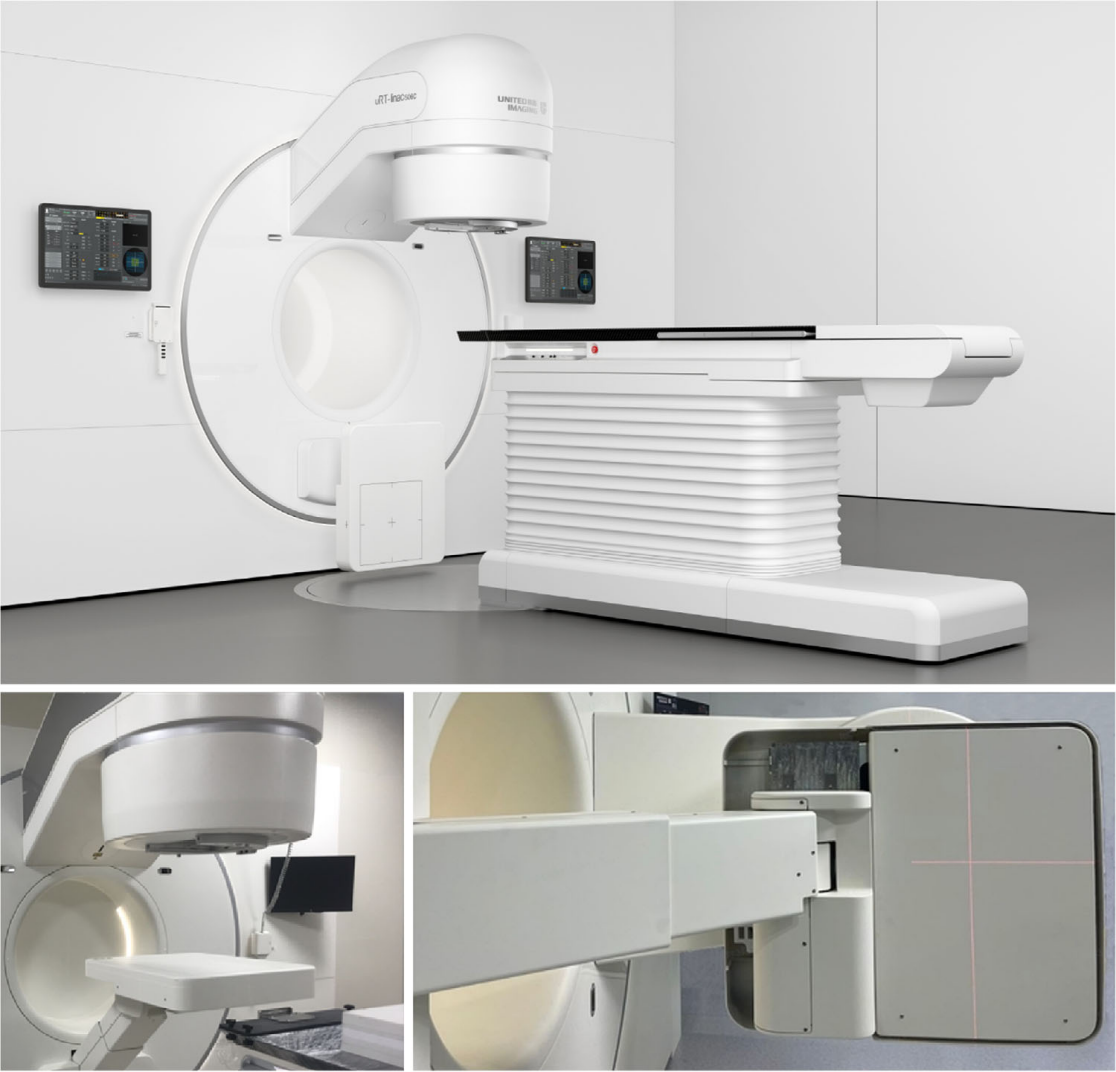
UIH uRT‐ linac 506c linear accelerator with integrated EPID system.

In recent years, a new medical linear accelerator model, UIH uRT‐linac 506c (United Imaging Healthcare Co., Ltd., Shanghai, China), was developed and implemented in clinics.^17^ UIH uRT‐linac 506c (Figure 1) can perform on‐board diagnosis‐level fan‐beam CT acquisition before and/or after treatment delivery and perform PSQA with integrated EPID system. UIH EPID Plan QA is an integrated module for plan verification, featuring a highly integrated system that eliminates many time‐consuming manual tasks, including measurement settings, plan delivery, collecting data, report printing, and batch data acquisition. A Monte Carlo‐based algorithm is used in this system for the prediction of dose distribution measured by EPID.^18^, ^19^, ^20^, ^21^, ^22^ The algorithm comprises several components including particle source, particle transport, and energy deposition modeling. Each randomly sampled particle, characterized by its unique type, energy, position, and trajectory, is accurately simulated until it enters the flat panel and generates a corresponding energy response. Compared to empirical or analytical approximation models, the Monte Carlo model provides a more precise description of the particle generation and energy deposition process, presenting advantages such as allowing accurate consideration of particles and precise simulation for the particle interaction, which makes this method a gold standard in dose calculation. However, it should be noted that statistical uncertainty plays an important role in the dose calculation accuracy. The application of the EPID system should be carefully managed.

TG‐307 emphasized the need for caution when using EPIDs for dosimetric verification, recognizing potential risks in model accuracy and calibration.^15^ Before clinical deployment, a PSQA system requires comprehensive validation testing to confirm its operational integrity. These tests should cover a range of delivery scenarios to assess the accuracy of the system and establish a benchmark for future maintenance and calibration. Testing aspects could include mechanical positioning, dose response, measurement linearity with dose, imaging quality, uniformity, and reproducibility. It is important to confirm that the portal imager and dosimetry system work together seamlessly to produce accurate dose measurements and analysis within clinically relevant settings. In general, before implementing a new EPID system in clinical practice, it is vital to validate this system in a way that covers all treatment modalities and energies that the system will encounter. This will ensure a comprehensive understanding of the operational dynamics and constraints of the system.

This study aimed to perform a general evaluation of a newly developed EPID system, comprising of Varex Imaging XRD 1642 EPID (Varex Imaging Corporation, Utah, USA) and EPID Plan QA module, on a UIH uRT‐ linac 506c linear accelerator for patient‐specific IMRT and VMAT QA tasks.

## 2 MATERIALS AND METHODS

### 2.1 Technical features

**FIGURE 2:**
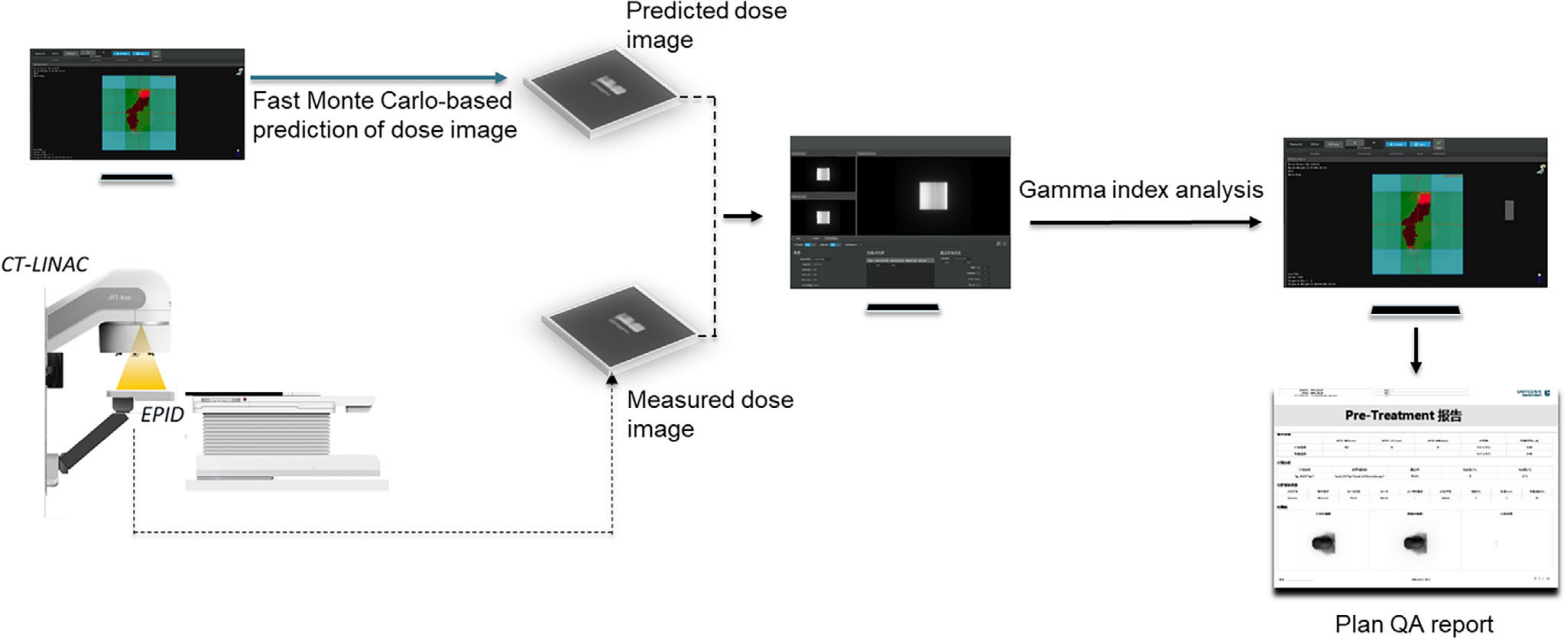
Pre‐treatment quality assurance workflow of UIH EPID Plan QA system.

All measurements were performed on a UIH uRT‐linac 506c (United Imaging Healthcare Co., Ltd., Shanghai, China) in the Huoshan County People's Hospital of Anhui Province. This advanced system seamlessly integrates a 16‐slice helical CT scanner (uCT 510, United Imaging Healthcare Co., Ltd., Shanghai, China) with a C‐arm linac, offering unprecedented capabilities in medical imaging and radiotherapy. The linac, which offers 6 MV photon beam, is equipped with a MLC comprised of 60 pairs of leaves. The MLC leaf widths are 5 mm for the central 40 pairs and 10 mm for the peripheral 20 pairs at the isocenter. A Varex Imaging XRD 1642 EPID (Varex Imaging Corporation, UT, USA) is installed on the UIH uRT‐linac 506c. Varex Imaging XRD 1642 is an a‐Si EPID with a 40.96 cm × 40.96 cm panel containing 1024×1024 pixel matrix yielding 0.4 mm pixel pitch. All EPID images were acquired with a source‐to‐detector distance of 100 cm and analyzed in EPID Plan QA module during this work. The uncertainty of Monte Carlo dose calculation was set to 0.5%. The general workflow of this EPID system for plan QA is demonstrated in Figure 2.

### 2.2 Quality assurance of the EPID

The quality assurance of an EPID system has been comprehensively described in AAPM TG‐58 and TG‐307 reports.^8^, ^15^ In order to validate an EPID system for patient imaging, several aspects of the EPID should be evaluated for the confirmation of its functionality as a dosimetry verification tool, such as dose response, field size dependence, uniformity, and reproducibility. Considering that this study aimed to perform a pre‐clinical assessment of the XRD 1642 EPID, several tests were selected per recommendations from the vendor to ensure this EPID will function accurately as a dosimetry verification tool. Generally, the system should go through a series of tests to ensure that it works as intended, which could include multiple delivery field patterns to verify the system and generate a baseline for future maintenance. The dosimetry system should consistently calculate the dose distributions correctly that fall within the clinical delivery requirement.

**FIGURE 3:**
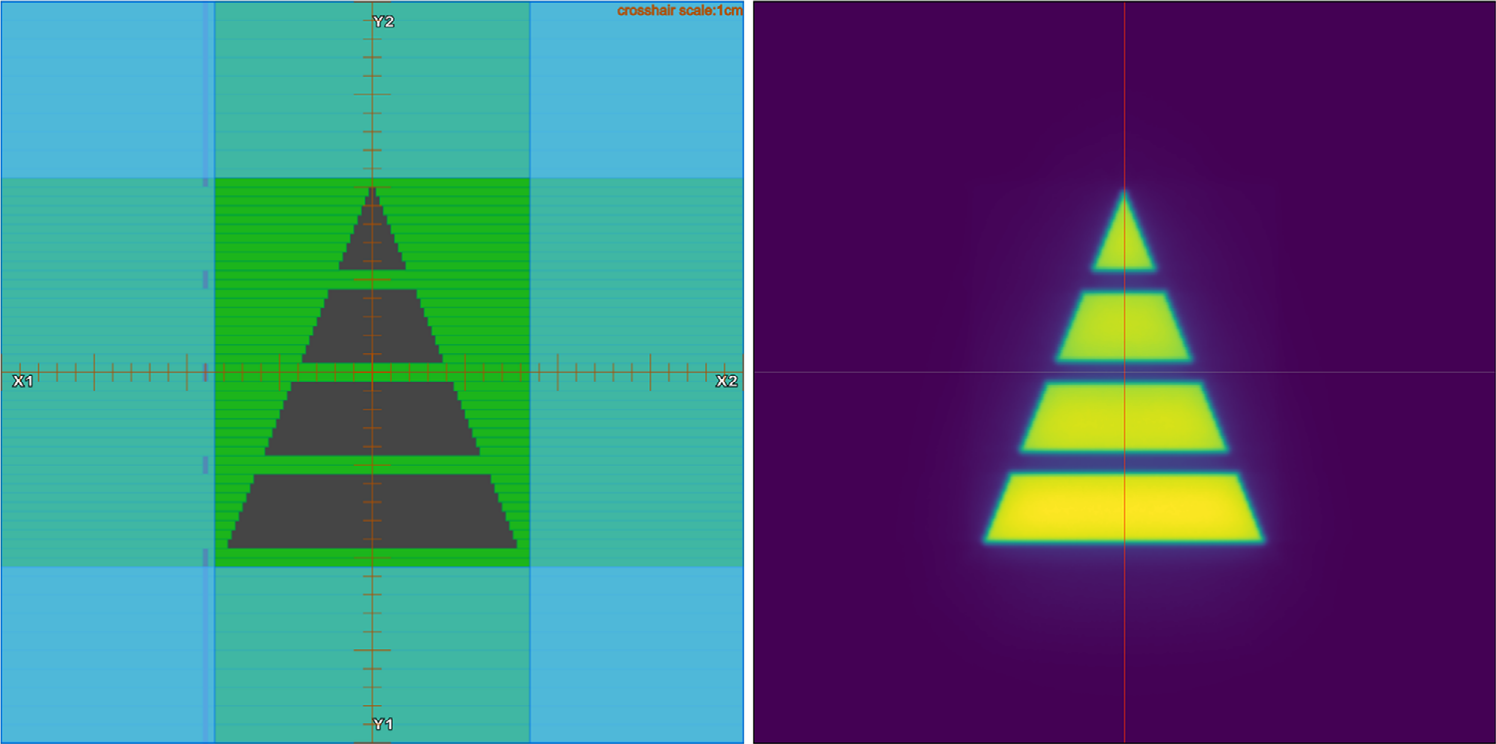
The pyramid field used for quality assurance of the EPID system. Left panel: demonstration of MLC positions of the pyramid field. Right panel: the measured EPID image for the pyramid field plan.

For simplicity, a static plan with one pyramid field, as demonstrated in Figure 3, was used for the tests designed to analyze the sensitivity of the EPID on the detection of errors. In addition, the picket fence test for the EPID system was also performed and the corresponding results are included as Figure S1 in the supplementary materials. A 1 mm MLC leaf shift in the right direction of was applied to the pyramid‐field plan, and the measured EPID image was compared with that of the original plan to test the sensitivity of the system to delivery discrepancies. The following changes were introduced to the pyramid‐field plan, and the results were analyzed by comparing the measured EPID images for the modified plans with the predicted EPID images for the original plan to assess the accuracy of the algorithm.

1. MLC offset. One random pair of MLC leaves involving forming the pyramid field was selected. The plan was modified by applying multiple shifts, that is, ± 1, ± 2, and ± 3 mm, to this leaf pair in the moving axis of the leaves. Dose profiles of the selected leaf pair would be extracted to measure the positional shift on the EPID image.
2. MU deviation. The MU count of the original plan, set at 200 MU, was modified by ± 1%, ± 2%, ± 3%, ± 4%, and ± 5%. The mean reading of pixels in a preset area at the center of the EPID image was used for the analysis. The difference between original and modified plans were calculated by comparing the mean readings.
3. Collimator angle. Collimator angle of the original plan was modified by ± 0.1°, ± 0.3°, ± 0.5°, ± 0.7°, ± 1.0°, ± 1.5°, and ± 2°. The angle between edges of pyramids in EPID images of the original plan and the modified plan was measured as a surrogate for the difference in collimator angles. An in‐house program was developed for the analysis of the collimator angle, utilizing the positional information of the left and right edges of the pyramid. Specifically, the program first identifies points along the two edges in the pyramid, then fits them into straight lines and converts them into inclination angles. After obtaining the inclination changes for each edge, the average value is taken as the final result.

### 2.3 Pre‐treatment validation of the EPID for PSQA

#### 2.3.1 Patients and treatment planning

A total of 16 patients with various tumor sites were included in this study. Plan CTs were acquired using the on‐board CT scanner of uRT‐linac 506c. Patients were divided into two groups, eight patients were enrolled for dynamic IMRT verification, and the other eight patients were enrolled for VMAT verification. Details regarding the tumor site and the treatment plan of each patient, including number of beams, are summarized in Table 1.

**TABLE 1:** Patient characteristics, planning details, and enrollment of induced error tests for pre‐treatment validation of the EPID system.

Patient no.	Planning technique	Tumor site	Number of beams	Induced errors				
				Collimator angle	Field offset	Field size	Monitor units	MLC functionality
1	IMRT	Rectum	7				✔	✔
2	IMRT	Rectum	7		✔	✔	✔	✔
3	IMRT	Cervix	9	✔	✔			
4	IMRT	Lung	5	✔	✔	✔		
5	IMRT	Brain	6			✔	✔	✔
6	IMRT	Oropharynx	9	✔	✔	✔		
7	IMRT	Nasopharynx	9	✔	✔	✔		
8	IMRT	Breast	6				✔	
9	VMAT	Pelvis	1	✔		✔	✔	
10	VMAT	Cervix	1		✔		✔	
11	VMAT	Breast	2	✔		✔		
12	VMAT	Lung	1		✔		✔	
13	VMAT	Abdomen	2		✔		✔	✔
14	VMAT	Head and neck	2	✔		✔		✔
15	VMAT	Neck	1		✔			✔
16	VMAT	Esophagus	2	✔		✔		✔

#### 2.3.2 Validation methodology

Before the EPID could be used in clinic, it must undergo a series of tests to validate the system for the intended purposes. Validation tests should include various delivery patterns to verify the integrity of the EPID and establish a baseline as a reference for future maintenance. These tests should be able to verify the portal imager is working correctly with the dosimetry system by consistently calculating the correct dose values with the correct geometrical dose distributions that fall within the expected clinical delivery parameters intended for the linac.

Creating plans and fields with induced errors can be used to determine the sensitivity of a system in error detection and confirm that the system matches expectations and specifications. In this study, two modalities including IMRT and VMAT were utilized for the validation of the system. For each modality, random patients were selected from the enrolled cohort for the validation of each feature. Upon completing the validation process, the user should have gained a comprehensive understanding of the performance features and constraints of the EPID and the corresponding analysis system.

Several induced error tests were performed to validate the EPID system for PSQA in clinical practice. The γ passing rates between the predicted and measured datasets would be compared with that of the original plan without the induced perturbation. Global γ index analysis was used for all comparisons between TPS reference datasets and EPID measured datasets using the criteria with 2% dose difference, 2 mm DTA, and 10% dose threshold.^17^, ^23^ The validation tests with induced errors or perturbations are listed as follows, and the patients randomly selected for each test has been indicated in Table 1.

1. MU accuracy, induced by increasing a certain percentage to the original MUs of the corresponding field. For each field, three levels of error were added to the MUs with 2.5%, 3.5%, and 4.5% increments, respectively. Four IMRT plans and four VMAT plans were selected for this test with 26 fields and 5 arcs in total, respectively.
2. Collimator angle error, induced by rotation the collimator by a certain angle. For each field, four rotation angles, that is, 1°, 2°, 3°, and 4°, were applied to the collimator. Four IMRT plans and four VMAT plans were selected for this test with 32 fields and 7 arcs in total, respectively.
3. Field offset, induced by shifting the corresponding field to the right direction by a certain distance. Four levels of lateral shifts, that is, 1.5, 2.5, 3.5, and 4.5 mm, were applied to each field. Five IMRT plans and four VMAT plans were selected for this test with 39 fields and 5 arcs in total, respectively.
4. Field size error, induced by expanding the MLC‐defined field through extending the leaf pairs. Three levels of field expansion, that is, 1, 2, and 3 mm, were applied by extending the leaf pairs in the both directions by half the intended distance. Five IMRT plans and four VMAT plans were selected for this test with 36 fields and 7 arcs in total, respectively.
5. MLC functionality error, induced by manually disabling one pair of MLC leaves. One random pair of MLC leaves were disabled successively for several times with separate measurements. Three IMRT plans and four VMAT plans were selected for this test with 20 fields and 7 arcs in total, respectively.

#### 2.3.3 ArcCHECK validation

The ArcCHECK (Sun Nuclear Corporation, Melbourne, Florida, USA) is a cylindrical diode array phantom designed for three‐dimensional dose verification, consisting of 1386 semi‐conductor diode detectors arranged in a helical pattern within an acrylic cylinder. In addition to the above tests, the ArcCHECK was used to validate the EPID system, including PSQA with a series of standardized plans and treatment plans, and induced error tests with a 10 cm × 10 cm open‐field static plan. The detailed methodology and results are included in the supplementary materials to avoid confusion with the above tests.

### 2.4 Statistical analysis

Pearson's correlation coefficient (*ρ*) is used to evaluate correlations between γ passing rate and induced errors or perturbations as an indication for the detection sensibility of the EPID. |*ρ*| ≥ 0.7 is considered a strong correlation, 0.5 ≤ |*ρ*| < 0.7 is considered a moderate correlation, 0.3 ≤ |*ρ*| < 0.5 is considered a weak correlation, and |*ρ*| < 0.3 is considered no correlation. A *p*‐value of 0.05 or lower is considered statistically significant. All statistical analyses were performed using IBM SPSS Statistics 26.0 software (IBM Corporation, Armonk, New York, USA).

## 3 RESULTS

### 3.1 Quality assurance

**FIGURE 4:**
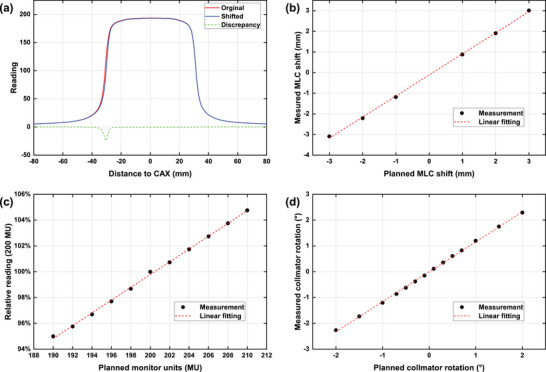
Quality assurance results of the EPID system. (a) Measured dose profiles before and after a 1 mm MLC leaf shift. (b) MLC offset test, (c) MU deviation test with readings shown relative to that of 200 MU, and (d) collimator angle test between measured and planned data.

Figure 4 shows the quality assurance results for the EPID system. Figure 4a displayed the measured dose profiles before and after a 1 mm MLC leaf shift to the pyramid‐field plan. Figure 4b–d compared the measured EPID images for the modified plans with the predicted EPID images for the original plan. For the MLC leaf offset in Figure 4b, the measured lateral shifts of the selected leaf pair are in good agreement with the planned value base on the dose profiles. The deviation of measured shifts from planned shifts are −0.09, −0.21, −0.18, −0.12, −0.10, and +0.01 mm from −3, −2, −1, 1, 2, and 3 mm, respectively. Measurements for other involving leaf pairs showed similar results. The MU deviation test results are shown in Figure 4c. The EPID accurately measured the MU count with trivial errors smaller than 0.25%. Technically, MU variation with a magnitude of 0.5% could be detected by the EPID. Figure 4d shows that the measured angles between pyramid edges are consistent with the corresponding differences in collimator angles.

### 3.2 Pre‐treatment validation for IMRT

**FIGURE 5:**
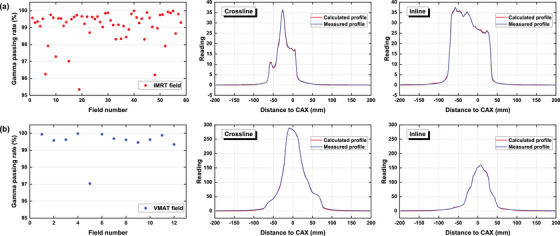
γ passing rates of the original IMRT (a) and VMAT (b) plans measured by the EPID system, as well as crossline and inline profiles of one random plan from each group.

Figure 5 displays the γ passing rates of the original IMRT and VMAT plans for all patients where no errors are introduced, as well as crossline and inline dose profiles of one random plan from each group for demonstration. The results indicate that the predicted and measured datasets are in good agreement, with all passing rates above 95%. Additionally, the profiles of all plans demonstrate a clear concurrence between the calculated and measured data.

**FIGURE 6:**
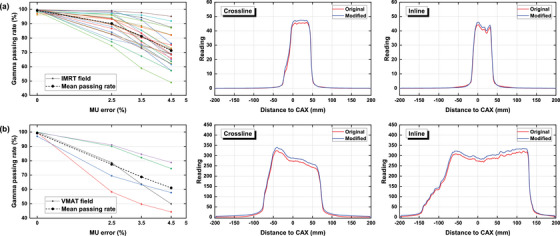
γ passing rates of the IMRT (a) and VMAT (b) fields with increasing MU error measured by the EPID system, as well as crossline and inline profiles of one random field from each group with a 4.5% MU increase.

**FIGURE 7:**
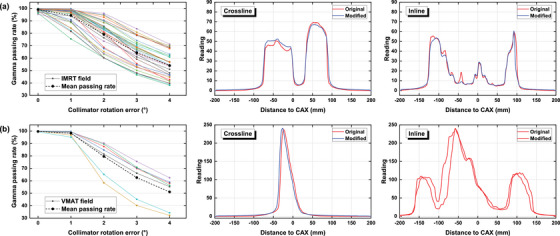
γ passing rates of the IMRT (a) and VMAT (b) fields with increasing collimator angle error measured by the EPID system, as well as crossline and inline profiles of one random field from each group with a 4° collimator angle increase.

**FIGURE 8:**
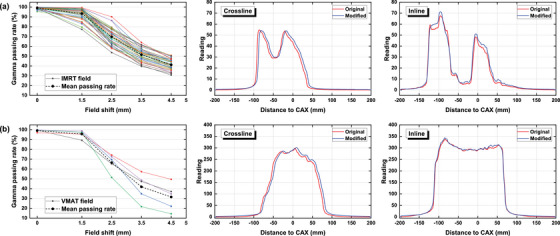
γ passing rates of the IMRT (a) and VMAT (b) fields with increasing field shift measured by the EPID system, as well as crossline and inline profiles of one random field from each group with a 4.5 mm field shift.

**FIGURE 9:**
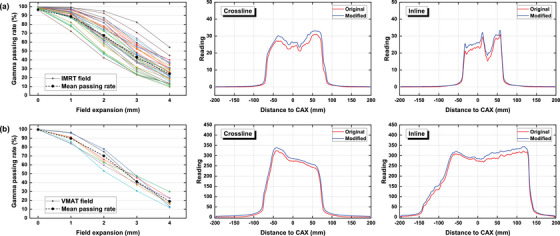
γ passing rates of the IMRT (a) and VMAT (b) fields with increasing field expansion measured by the EPID system, as well as crossline and inline profiles of one random field from each group with a 4 mm field expansion.

**FIGURE 10:**
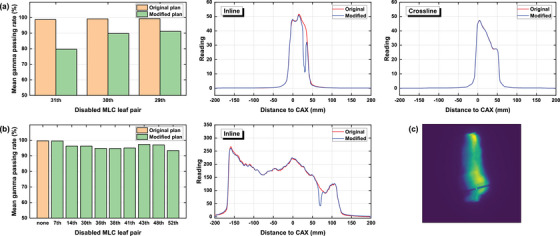
Mean γ passing rates of original and modified IMRT (a) and VMAT (b) fields with one disabled MLC leaf pair measured by the EPID system, as well as profiles of one random field from each group. (c) The EPID image for a modified VMAT field.

Figures 6, 7, 8, 9, 10 demonstrate the variation of γ passing rates (2%/2 mm, 10% low‐dose threshold) of IMRT and VMAT fields with induced errors, as well as examples of measured crossline and inline profiles for each modality. Examples of γ evaluation images were included as Figure S2 in the supplementary materials. The γ passing rate showed a significantly decreasing pattern with induced errors concerning MU accuracy, collimator angle, field offset and field size. The mean γ passing rate, represented by the dashed line, was found to be strongly correlated with the magnitude of the induced error. For the MLC functionality error, it can be seen from the figures that the impact of the disabled leaf pair varied depending on its relative position in the field.

Based on the crossline and inline dose profiles, it is evident that the increased dosimetric discrepancy between predicted and measured datasets differed depending on the type of induced error. Collimator angle error caused a distortion on the dose distribution. Field offset caused a lateral shift on the dose distribution demonstrated by the measured crossline profile. Field size error caused a general enlargement of the radiation field leading to increased height and width on both crossline and inline profiles. MU error caused an increased height on both crossline and inline profiles. MLC functionality error induced by disabling one leaf pair caused a pit on the corresponding position of the incline profile.

## 4 DISCUSSION

The incorporation of EPIDs has transformed treatment verification and QA processes, underscoring the transition toward more precise and personalized cancer treatments. Prior researches have elucidated various EPID‐based QA methodologies.^5^, ^24^ The enhanced accuracy and efficiency of EPID‐based PSQA can potentially lead to improved patient outcomes by ensuring the precision of dose delivery. The adoption of QA system demands considerations regarding the ability of this system to capture complex dosimetric information with high spatial resolution. The current work detailed a pre‐clinical PSQA validation process to test the EPID Plan QA module featuring a Monte Carlo‐based dose prediction algorithm coupled with a Varex imaging XRD 1642 EPID. The testing results proved the ability of this system.

Research efforts have been dedicated to EPID predictive model development for PSQA. Monte Carlo simulation method was leveraged to enhance the accuracy of dose calculations and predictions.^25^, ^26^, ^27^, ^28^, ^29^ Siebers et al. introduced a Monte Carlo computation method for dosimetric a‐Si EPID images and demonstrated the feasibility of using Monte Carlo simulations to accurately predict the dosimetric properties of EPID images, laying the groundwork for subsequent developments in EPID‐based dosimetry.^25^ Parent et al. compared an a‐Si EPID calibration method based on Monte Carlo prediction of response, suggesting that Monte Carlo‐based calibration could enhance the dosimetric application of EPIDs by improving the accuracy of dose measurements.^26^ Wang et al. developed a Monte Carlo‐based adaptive EPID dose kernel that accounts for the varying field size responses of imagers, allowing for more precise dose calculations, especially in scenarios with complex field sizes and shapes.^27^ Such advancements underscore the potential for Monte Carlo‐based algorithms to refine the correlation between predicted and measured doses, thus improving the reliability of treatment plans before their clinical application. The EPID system evaluated in the current work features a Monte Carlo based algorithm for dose image prediction and automated PSQA with minimal manual operations. Although several studies were devoted to this subject as detailed above, there has not been researches on the pre‐treatment evaluation of a commercially available system to our knowledge.

Prior to application for PSQA, the EPID system underwent a QA process with static‐field plans to measure and validate its integrity. Three types of induced errors were used for the process, including MLC offset, MU deviation, and collimator angle. Results showed that the EPID is working correctly with the dosimetry system. Typically, a γ passing rate of over 95% with 3%/2 mm criteria and a 10% dose threshold is the standard criteria as recommended for intensity modulated plans in TG‐218 report.^23^ Results showed that γ passing rates of all IMRT and VMAT plans in this work were well within the expectation regarding the tolerance level for PSQA, on par with PSQA measurement results reported in the literature for various models of EPIDs.^24^, ^30^, ^31^, ^32^

There are several challenges in the clinical implementation of EPIDs, including rigorous commissioning, calibration, and validation protocols​​. The methodology of this work adheres to existing guidelines,^9^, ^15^, ^23^ ensuring that the performance of the EPID system in detecting induced errors is robust and clinically relevant. As mentioned above, comparative measurements using plans with induced errors can be used to test the sensitivity of a system in error detection. In this work, modified IMRT and VMAT plans with induced errors were used to verify the sensitivity of the EPID in error detection. Each induced error was applied to plans and fields with several magnitudes. The resulting γ passing rate of the modified plans were then compared with that of the original plans with no errors.

Five types of machine errors that are most likely to affect the acquisition results of the EPID were tested in this work, that is, MU count, collimator angle, field offset, field size, and MLC functionality errors. To measure the sensitivity of the EPID to these errors objectively, random patient plans for each machine error were selected from the enrolled patients. All the modified plans were imported into the EPID Plan QA module, and the corresponding images were collected automatically using the integrated batch acquisition function. One batch acquisition task took only about 7 min of manual operation. Once the data acquisition was complete, the predicted and measured datasets were automatically compared and analyzed to obtain the γ passing rates for all the imported plans, and the corresponding QA report could be generated concurrently. For each series of tests with induced errors, the EPID system showed satisfying measurement accuracy and promising analysis results.

In addition, the ArcCHECK phantom was used to validate the EPID system, including PSQA with treatment plans, and induced error tests with a 10 cm × 10 cm open‐field static plan, as shown in the supplementary materials. For PSQA, a series of standardized test cases per recommendations from AAPM TG‐119 report^33^ and several patient plans including IMRT and VMAT were used in this study. In general, no meaningful differences were found between EPID and ArcCHECK in either IMRT and VMAT plans. For induced error tests, EPID appeared higher or at least similar sensitivity to errors including MU reduction, jaw offset, MLC‐field size expansion, MLC‐field offset, and collimator rotation compared with ArcCHECK. Based on the current results, the clinical acceptance or tolerance level could be decided based on AAPM TG‐218 and TG‐307 reports and institutional standard.^15^, ^23^

Several studies have demonstrated the application of induced error test method on the EPID QA. Fredh et al. tested four plans with MU, MLC, and collimator errors using the Epiqa system (EPIdos, Ivanka pri Dunaji, Slovakia).^34^ Wu et al. tested 10 plans with MLC and gantry errors using the RadCalc EPID system (LAP, Boynton Beach, Florida, USA).^24^ Bresciani et al. tested one plan with MU and MLC errors using the Portal Dosimetry system (Varian Medical Systems, Palo Alto, California, USA) and the PerFRACTION system (Sun Nuclear.Melbourne, Florida, USA).^35^ The data from each study provided a deeper insight into the capabilities and limitations of EPID‐based QA systems and methodologies.^36^, ^37^, ^38^ While all systems could detect significant errors, the sensitivity to smaller, clinically relevant deviations varied, emphasizing the need for robust QA systems capable of identifying subtle inaccuracies.

As treatment techniques continue to evolve towards increasingly sophisticated and personalized approaches, the role of precise and reliable QA systems becomes ever more critical. The results of this study suggest this EPID system could play a role in this evolving landscape, aiding in the validation of emerging treatment techniques and contributing to the overarching goal of enhancing therapeutic efficacy while minimizing adverse effects. While the study confirms the utility of this EPID in PSQA, it also illuminates the inherent challenges, such as sensitivity to calibration procedures and the application for other modalities. Besides, for a new EPID to be implemented in clinic, it is recommended that the commissioning of the attached linear accelerator should be performed before the EPID system could be used for PSQA. Although this process was completed for this work, it is not included in this paper for conciseness. Future researches could include expanding its applicability across diverse beam types and treatment modalities, and exploring its integration with treatment adaptation processes.

## 5 CONCLUSION

This work illustrates the effective utilization of a Monte Carlo‐based EPID Plan QA system, coupled with Varex Imaging XRD 1642, in PSQA for IMRT and VMAT. Through rigorous testing against a spectrum of items with introducing error, including MU, collimator angle, field offset, field size, and MLC functionality, this study demonstrates the proficiency of this EPID system in detecting dosimetric discrepancies with high accuracy, underpinning its potential as a robust tool for ensuring precise and reliable radiotherapy. Future endeavors should focus on refining the system by exploring their integration with emerging treatment modalities, and validating their efficacy in diverse clinical scenarios.

## AUTHOR CONTRIBUTIONS

Conceptualization and methodology: Yanfang Liu, Zhi Wang, Tianlong Ji and Huidong Wang. Investigation and data curation: Xiaohe He, Li Xiao, Yanfang Liu, Zhi Wang and Tianlong Ji. Visualization: Wuji Sun, Chao Ge, Yinghua Shi, Xiaohe He and Li Xiao. Supervision: Yanfang Liu, Zhi Wang, Tianlong Ji and Huidong Wang. Writing—original draft: Wuji Sun. Writing—review & editing: All authors.

## CONFLICT OF INTEREST STATEMENT

Xiaohe He, Li Xiao, and Yanfang Liu are employed by United Imaging Healthcare Co., Ltd. The remaining authors have no relevant conflicts of interest to disclose.

## Supporting information



Supporting Information

Supporting Information
