# Supplementary material for: Evaluation of a Monte Carlo based EPID system for patient‐specific IMRT and VMAT quality assurance
Source: J Appl Clin Med Phys. 2025 Jul 15;26(7):e70178. doi: 10.1002/acm2.70178 (PMC12260258; doi:10.1002/acm2.70178)
Supplement: Supplementary file 1 — Supporting Information [file ACM2-26-e70178-s002.docx]

**S1. Supplementary figures for EPID tests**


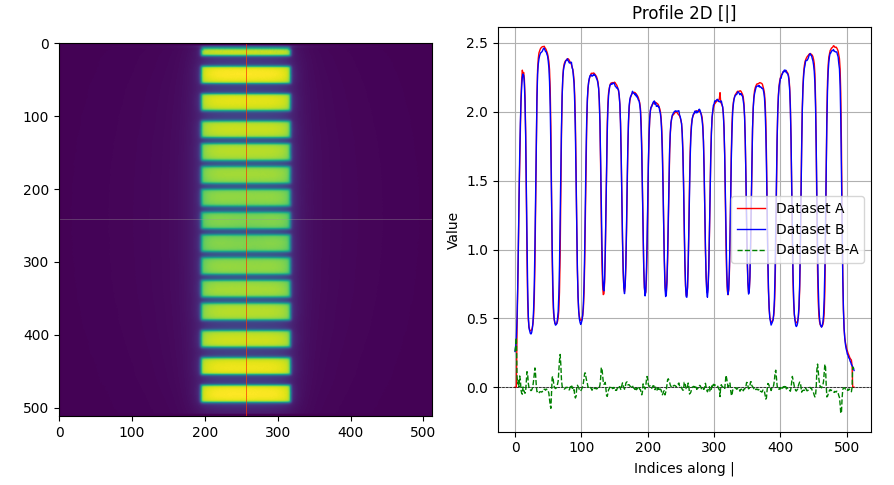


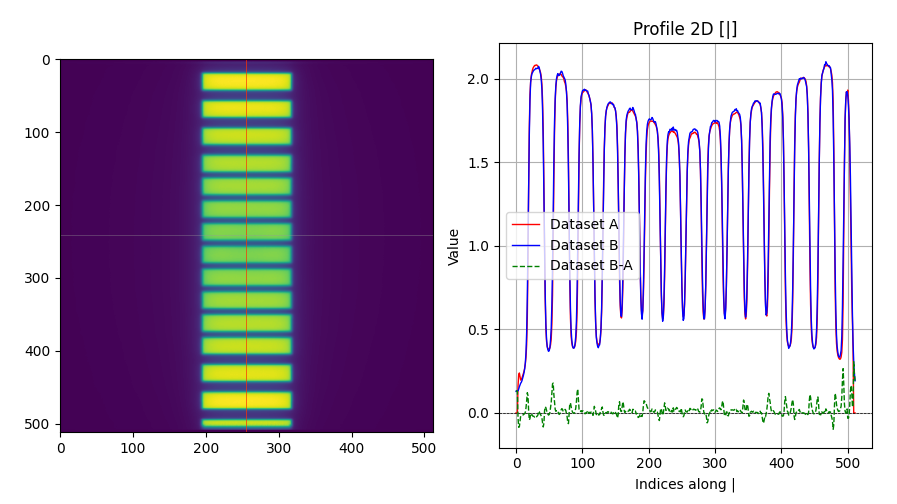

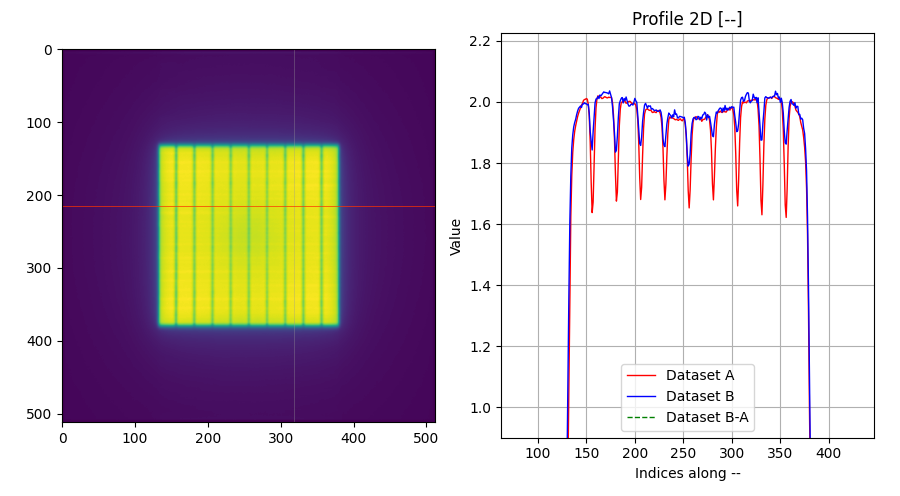


**Figure S1.** Picket fence tests for the EPID system and profile comparison between TPS predicted (blue) and EPID measured (red) datasets. The green dashed curve indicates the difference between red and blue curves.


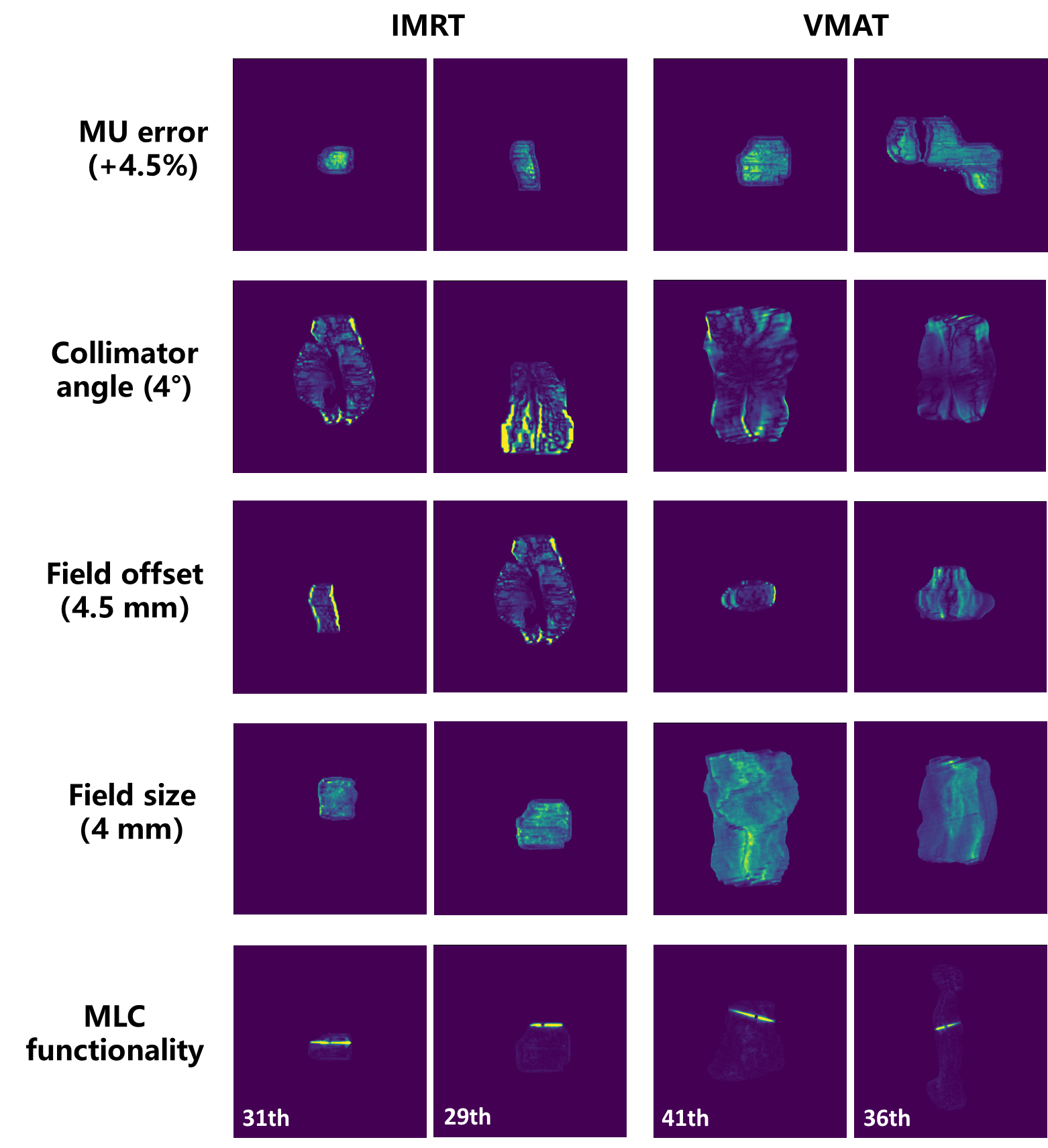


**Figure S2.** Examples of γ evaluation images between TPS and EPID datasets, including IMRT and VMAT field analyses for each induced error test.

**S2. ArcCHECK Validation**

**S1.1 Materials and methods**

For PSQA, a series of standardized test cases and several additional patient plans were used for measurements. The standardized test cases included multitarget (MT), prostate (PR), head/neck (HN), and two C-shape (C1 and C2) cases per recommendations from AAPM TG-119 report. Both IMRT and VMAT plans were tested for each case. Patient plans included five IMRT plans and three VMAT plans. The above plans were delivered on the ArcCHECK phantom positioned on the treatment couch with its center aligned with the isocenter. Same plans were measured using the EPID system for direct comparison.

For induced error tests, similar test methods were applied on the ArcCHECK. To simplify the comparison, a 10 cm × 10 cm open-field static plan was used for induced error tests. The induced errors included MU reduction, jaw offset, MLC-field size, MLC-field offset, and collimator angle. It should be noted that in MU reduction, jaw offset, and collimator angle tests, the 10 cm × 10 cm field was formed with jaws, while in MLC-field size and MLC-field offset tests, the field was formed with MLC to properly perform the corresponding tests.

The magnitudes of the induced errors are shown in the following section. The measured datasets were compared with the planned datasets using γ analysis with 3%/3 mm and 2%/2 mm criteria with a 10% low-dose threshold.

**S1.2 Results and discussion**

The PSQA results presented in Table S1 compared γ passing rates between the EPID system and ArcCHECK for IMRT and VMAT plans, using both 3%/3mm and 2%/2mm criteria with a 10% low-dose threshold. In general, no meaningful differences were found between EPID and ArcCHECK for either modality or gamma criteria. The high passing rates indicate excellent agreement between planned and measured datasets and comparable performance for these two systems.

**Table S1.** Mean (± standard deviation) γ passing rate comparison between EPID and ArcCHECK for IMRT and VMAT QA.

| **Plan** | **EPID γ passing rate (%, mean ± SD)** | | **ArcCHECK γ passing rate (%, mean ± SD)** | |
| --- | --- | --- | --- | --- |
|  | **3%/3mm** | **2%/2mm** | **3%/3mm** | **2%/2mm** |
| IMRT | 99.99 ± 0.01 | 99.19 ± 1.25 | 99.31 ± 0.85 | 98.21 ± 1.95 |
| VMAT | 100.00 ± 0.00 | 99.60 ± 0.50 | 99.88 ± 0.19 | 99.45 ± 0.34 |

Table S2 presents γ passing rates for a 10 cm × 10 cm open-field static plan with induced errors, comparing EPID and ArcCHECK under the same gamma criteria. Five error types were tested including MU reduction, jaw offset, MLC-field size expansion, MLC-field offset, and collimator rotation, with varying magnitudes as indicated in Table S2. Pearson’s correlation coefficients (ρ) were calculated to assess the relationship between induced error magnitudes and γ passing rates, and strong correlations were found for all sets of data, except for ArcCHECK with 3%/3mm in field size tests since γ passing rate maintained at 100%. Figure S3 showed examples of γ evaluation images and profiles comparison of the EPID system and ArcCHECK for each test.

For the baseline plan, both systems achieved near-perfect passing rates. As the magnitudes of each type of errors increased, EPID’s passing rates generally dropped sharply, while ArcCHECK maintained higher rates for both criteria in most cases. EPID appeared higher or at least similar sensitivity to MU reduction, jaw offset, MLC-field size expansion, MLC-field offset, and collimator rotation compared with ArcCHECK.

In general, the EPID system matches ArcCHECK’s performance for PSQA and offers superior sensitivity in certain errors, making it a reliable choice for clinical use.

**Table S2.** γ passing rate comparison between EPID and ArcCHECK for 10 cm × 10 cm open-field plan with induced error tests.

| **Error type** | **Induced error** | **EPID γ passing rate (%, mean ± SD)** | | **ArcCHECK γ passing rate (%, mean ± SD)** | |
| --- | --- | --- | --- | --- | --- |
|  |  | **3%/3mm** | **2%/2mm** | **3%/3mm** | **2%/2mm** |
| MU reduction | None (200 MU) | 100.00 | 100.00 | 100.00 | 99.60 |
|  | -2.5% (195 MU) | 88.00 | 35.45 | 96.20 | 86.90 |
|  | -3.5% (193 MU) | 37.10 | 33.95 | 88.20 | 78.50 |
|  | -4.5% (191 MU) | 35.06 | 32.53 | 79.70 | 76.40 |
| Jaw offset | None | 100.00 | 100.00 | 100.00 | 100.00 |
|  | X1 Jaw 1 mm expansion | 100.00 | 100.00 | 100.00 | 100.00 |
|  | X1 Jaw 2 mm expansion | 100.00 | 95.92 | 100.00 | 98.30 |
|  | X1 Jaw 3 mm expansion | 96.02 | 91.72 | 100.00 | 97.10 |
|  | X1 Jaw 4 mm expansion | 91.96 | 90.30 | 98.40 | 92.40 |
| MLC-field size | None | 100.00 | 100.00 | 100.00 | 100.00 |
|  | 1 mm expansion | 100.00 | 99.60 | 100.00 | 100.00 |
|  | 2 mm expansion | 100.00 | 97.62 | 100.00 | 100.00 |
|  | 3 mm expansion | 99.97 | 95.50 | 100.00 | 98.70 |
|  | 4 mm expansion | 98.22 | 92.37 | 100.00 | 94.50 |
| MLC-field offset | None | 100.00 | 100.00 | 100.00 | 100.00 |
|  | 1 mm translational shift | 100.00 | 100.00 | 100.00 | 100.00 |
|  | 2.5 mm translational shift | 99.91 | 88.50 | 92.10 | 87.90 |
|  | 3.5 mm translational shift | 89.51 | 81.15 | 90.90 | 84.70 |
|  | 4.5 mm translational shift | 82.73 | 75.79 | 86.50 | 82.80 |
| Collimator rotation | None | 100.00 | 100.00 | 100.00 | 100.00 |
|  | 1° counterclockwise | 100.00 | 100.00 | 100.00 | 99.20 |
|  | 2° counterclockwise | 100.00 | 99.07 | 99.60 | 98.30 |
|  | 3° counterclockwise | 99.87 | 94.96 | 98.80 | 94.20 |
|  | 4° counterclockwise | 98.45 | 88.34 | 97.10 | 90.20 |


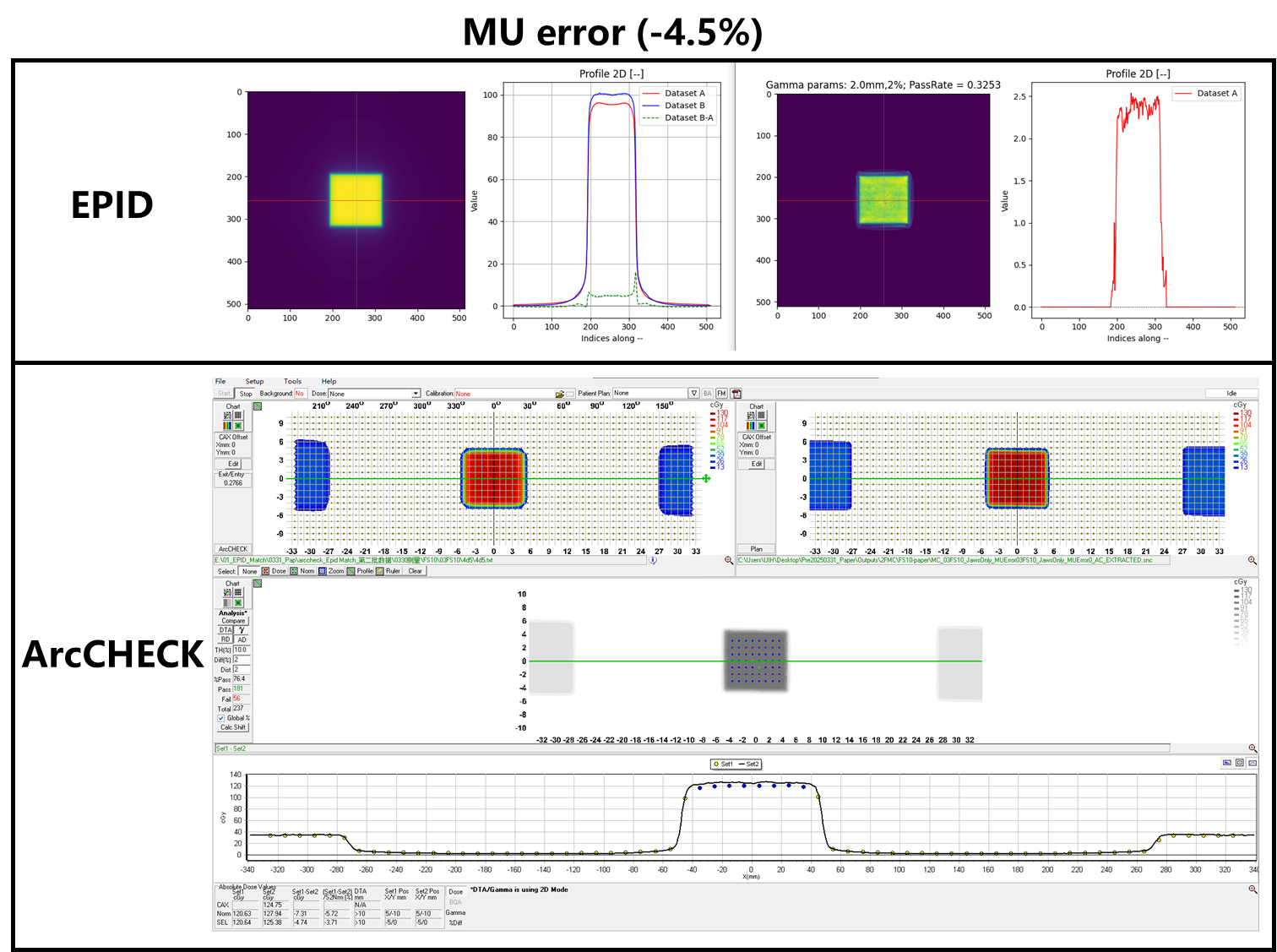


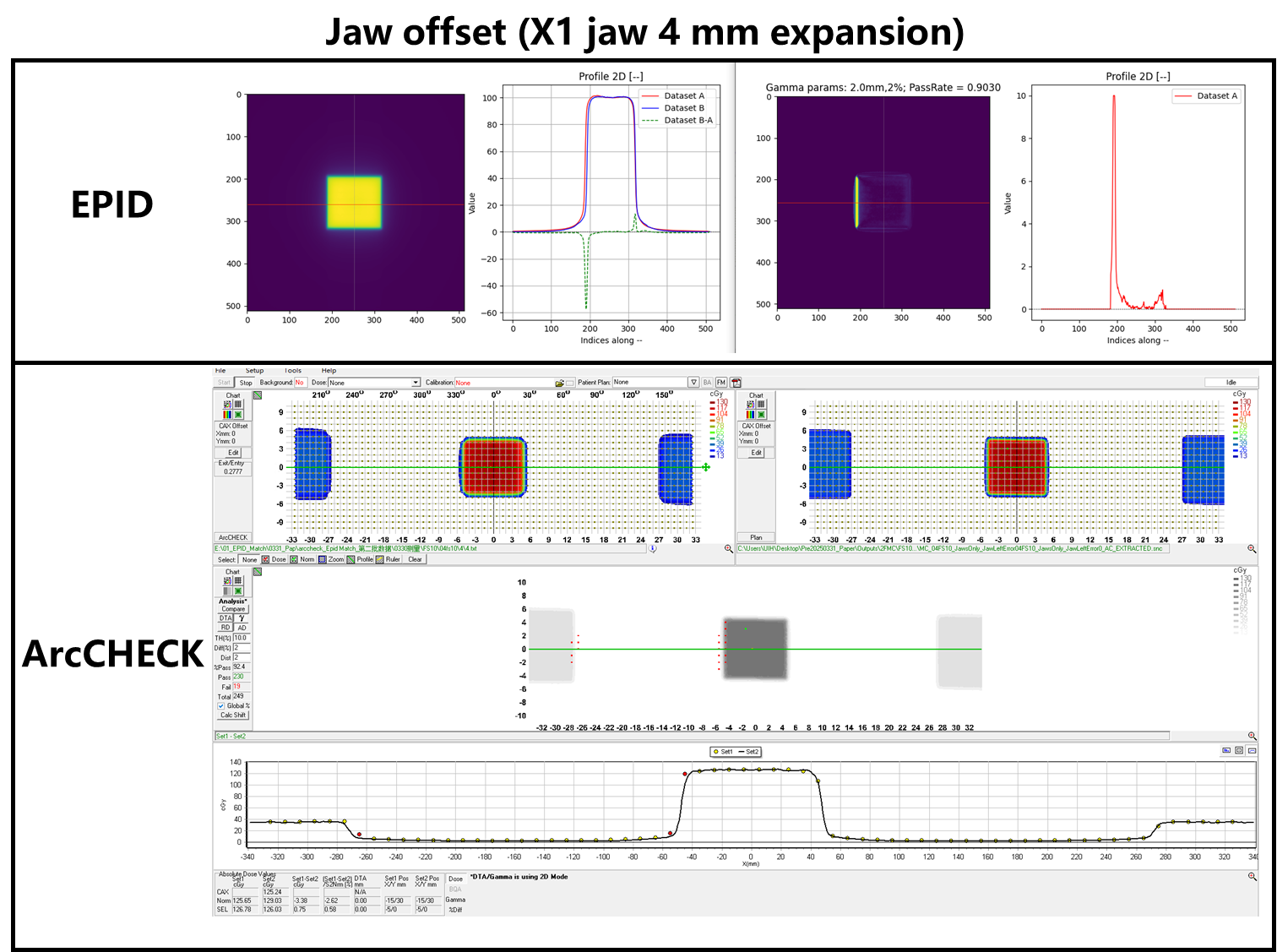


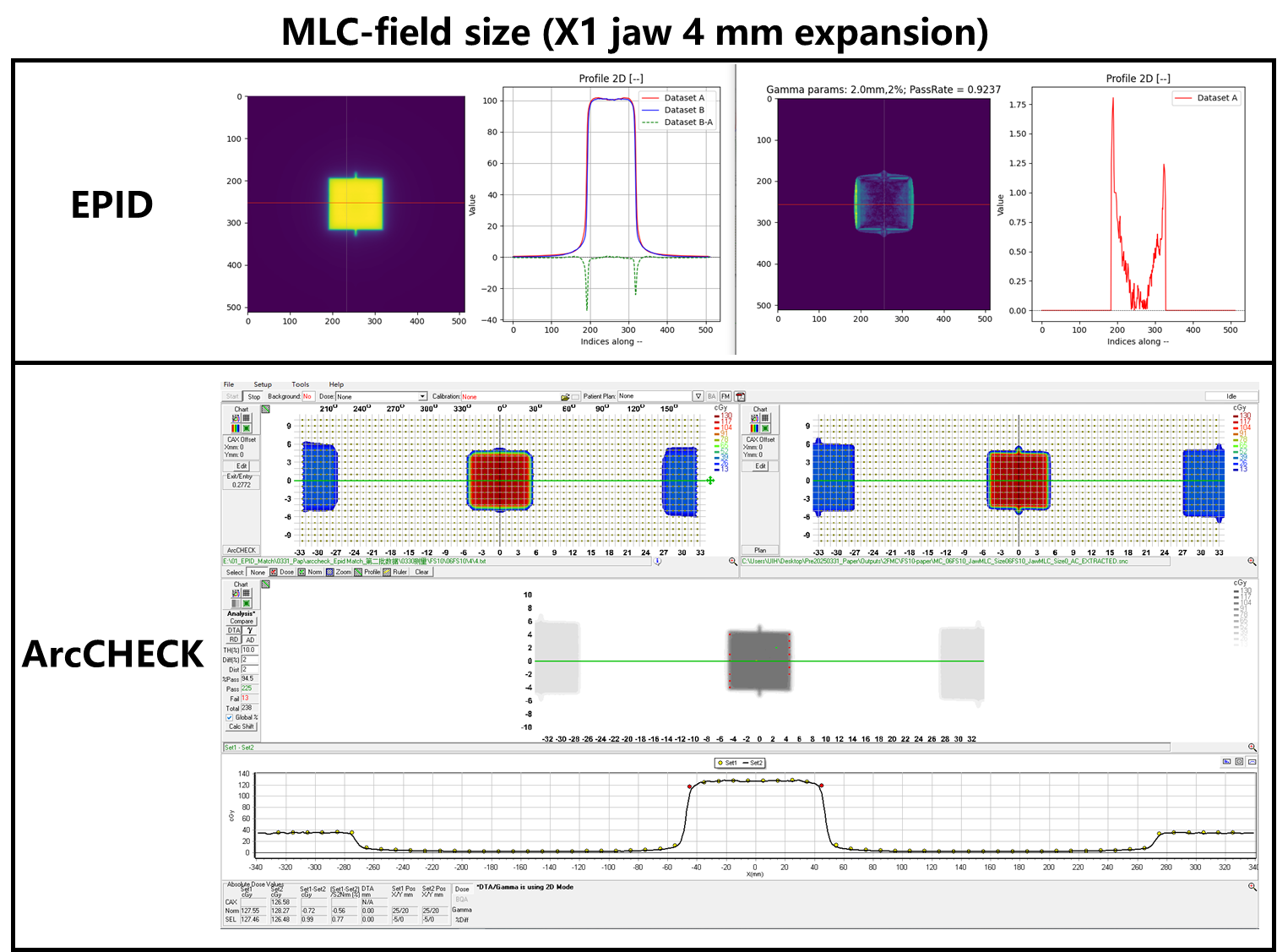


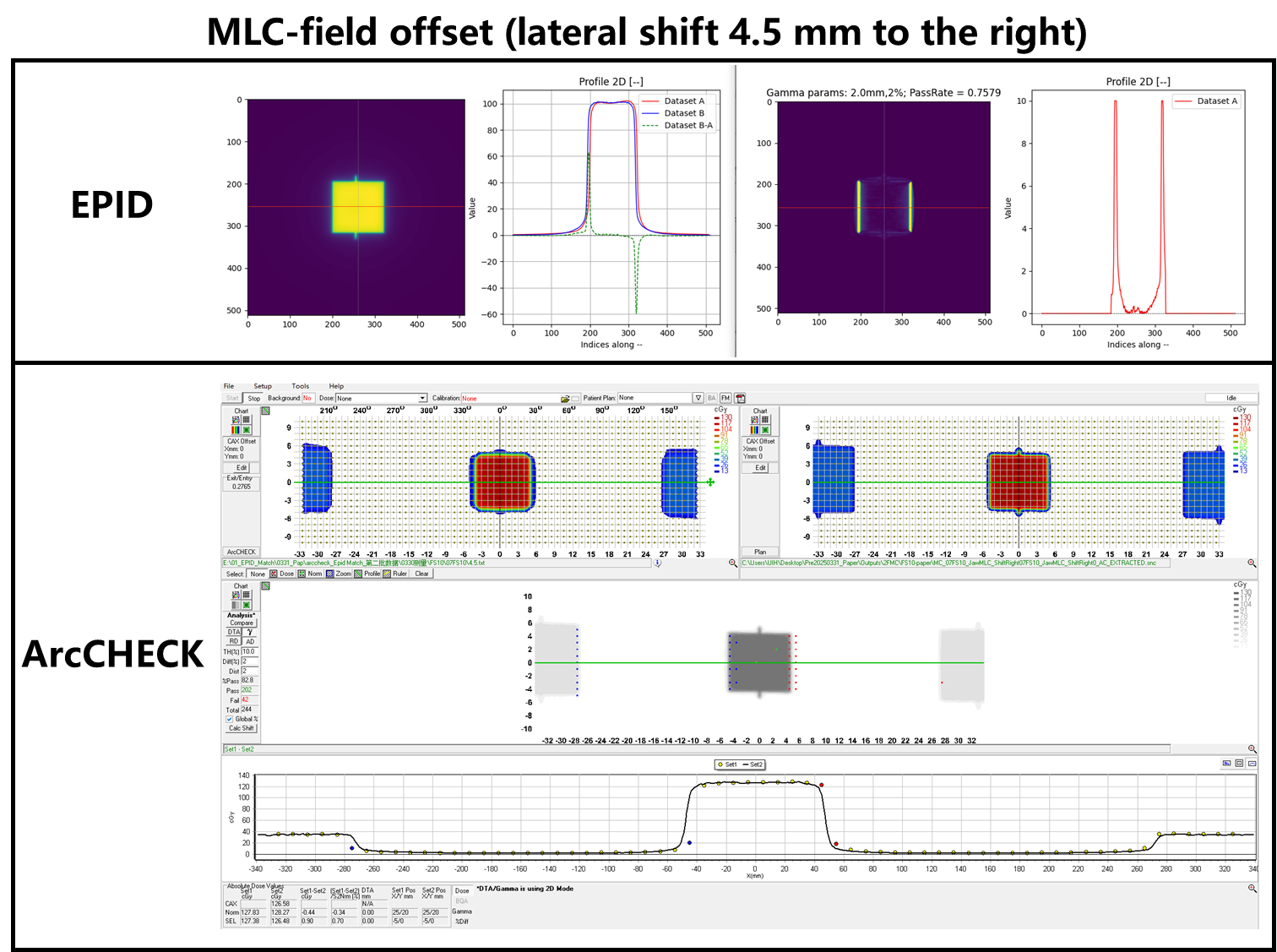


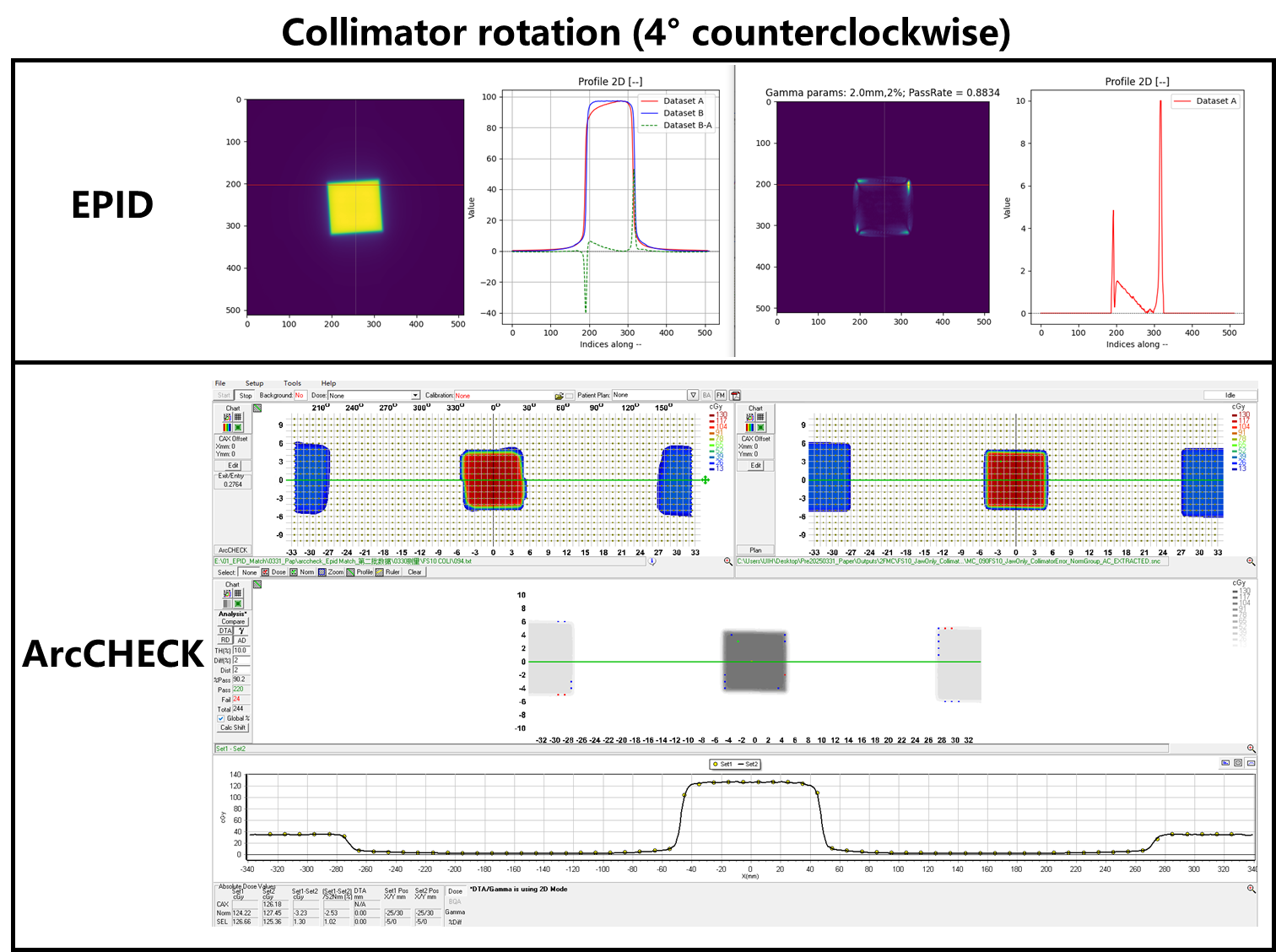


**Figure S3.** Examples of γ evaluation images between TPS predicted and measured datasets with EPID and ArcCHECK for each induced error test. Upper left panel (for each test): the red curve (Dataset A) indicates the EPID measured dataset with induced error, the blue curve (Dataset B) indicates the TPS predicted dataset without induced error, and the green dashed curve indicates the difference between Datasets A and B. Upper right panel: the red curve indicates the γ index.
